# Reply: Bayesian analysis supports the role of neoadjuvant chemoradiation followed by surgery for resectable locoregional esophageal cancer

**DOI:** 10.1111/1759-7714.14651

**Published:** 2022-09-14

**Authors:** Yongxing Bao, Yang Wang, Zhouguang Hui

**Affiliations:** ^1^ Department of Radiation Oncology, National Cancer Center/National Clinical Research Center for Cancer/Cancer Hospital Chinese Academy of Medical Sciences and Peking Union Medical College Beijing China; ^2^ Department of VIP Medical Services, National Cancer Center/National Clinical Research Center for Cancer/Cancer Hospital Chinese Academy of Medical Sciences and Peking Union Medical College Beijing China

To the editor:

Thank you for Rizzo's letter for discussion.^1^

The conceptual framework for network meta‐analysis (NMA), if properly applied, does not inflate type I and type II error (or false positive and false negative conclusion) by itself. However, similar to pairwise meta‐analysis, the challenges still present and even increase due to the complexity of network structure. Transitivity, consistency, and bias caused by publication are difficult to address. Therefore, the team agrees that the error is inherent in the research and should be evaluated and interpreted carefully in either frequentist or Bayesian framework.

Compared with NMA under the frequentist framework, the advantage of Bayesian NMA is that it takes into account the uncertainty through the posterior distribution, making the result more reliable. It is particularly useful with a small sample size, which is suitable for our study (the neoadjuvant radiotherapy group only contains 150 patients).

To echo Dr Rizzo, large‐scale, well‐designed clinical trials, as the golden standard, are still needed to confirm the findings and provide more insight by directly comparing the effectiveness of neoadjuvant chemotherapy and chemoradiotherapy for esophageal cancer.

## CONFLICT OF INTEREST

None.
